# Weight and Abdominal Pressure-Induced Shunt Trouble in Patients With Shunted Normal Pressure Hydrocephalus: A Comprehensive Study on Pressure Environment of Shunt System

**DOI:** 10.3389/fneur.2022.882757

**Published:** 2022-05-23

**Authors:** Masatsugu Kamo, Yoshinaga Kajimoto, Tomohisa Ohmura, Masahiro Kameda, Adam Tucker, Hiroji Miyake, Masahiko Wanibuchi

**Affiliations:** ^1^Department Neurosurgery, Osaka Medical and Pharmaceutical University, Takatsuki, Japan; ^2^Department Neurosurgery, Nishinomiya Kyoritsu Neurosurgical Hospital, Nishinomiya, Japan; ^3^Department Neurosurgery, Kitami Red Cross Hospital, Kitami, Japan; ^4^Department Neurosurgery, Nishinomiya Kyoritsu Rehabilitation Hospital, Nishinomiya, Japan

**Keywords:** normal pressure hydrocephalus (NPH), shunt malfunction, intra-abdominal pressure (IAP), intracranial pressure (ICP), hydrocephalus–surgery

## Abstract

**Objectives:**

We identified a new type of shunt malfunction (SM) in patients with normal pressure hydrocephalus (NPH). It is induced by weight change and can be treated with valve readjustment. There were two types of SM as follows: Underdrainage induced by the weight gain and overdrainage induced by the weight loss. This study aims to elucidate this mechanism by assessing the shunt pressure environment.

**Methods:**

The total pressure environment of the shunt system was prospectively studied in patients with shunted NPH at Osaka Medical College Hospital from 1999 to 2005. We measured the pressure environment during the initial pressure setting of the valve by the intracranial pressure (ICP) guide, after setting the valve, and when SM was suspected. We evaluated ICP, intra-abdominal pressure (IAP), and hydrostatic and perfusion pressures of the shunt system in the sitting and supine positions. The target ICP for valve setting was empirically set at the range from −8 to −13 mm Hg in the sitting position, referring to the external auditory meatus. During the study period, we identified five cases of SM induced by weight change and assessed the changes in the pressure environment across pre-SM, SM, and post-SM.

**Results:**

In four cases of underdrainage, gait disturbance worsened with an average weight gain of 6.8 ± 1.2 kg. With weight gain, IAP and ICP increased by 8.8 ± 1.6 and 4.8 ± 1.0 mm Hg, respectively. Consequently, ICP increased to −6.5 ± 1.9 mm Hg. One overdrainage patient developed an asymptomatic chronic subdural hematoma (CSDH) with a weight loss of 10 kg. With the weight loss, both IAP and ICP decreased by 5 mm Hg, and concomitantly, ICP decreased to −18 mm Hg. In all patients, the valve readjustment restored their ICP to the target pressure. After the valve readjustment, the gait disturbance improved immediately, and the CSDH disappeared after 1 month.

**Conclusion:**

In patients with shunts, the weight change was linked to ICP *via* IAP. Due to the weight change, the underdrainage occurred when ICP was above the target pressure, and the overdrainage occurred when ICP was below it. We named this SM as the weight and abdominal pressure-induced shunt trouble. The patients with SM along with weight changes should be the first to be tried for the valve readjustment.

## Introduction

The patients with shunt malfunction (SM) along with hydrocephalus is the primarily associated with mechanical obstruction of the valves and catheters (1). In the present study, we have identified a new type of functional SM that is caused by weight change and can be treated by readjusting valve pressure. This study presents that the weight change affected the intra-abdominal pressure (IAP) and caused SM by altering the intracranial pressure (ICP) linked to the IAP.

**Figure 1:**
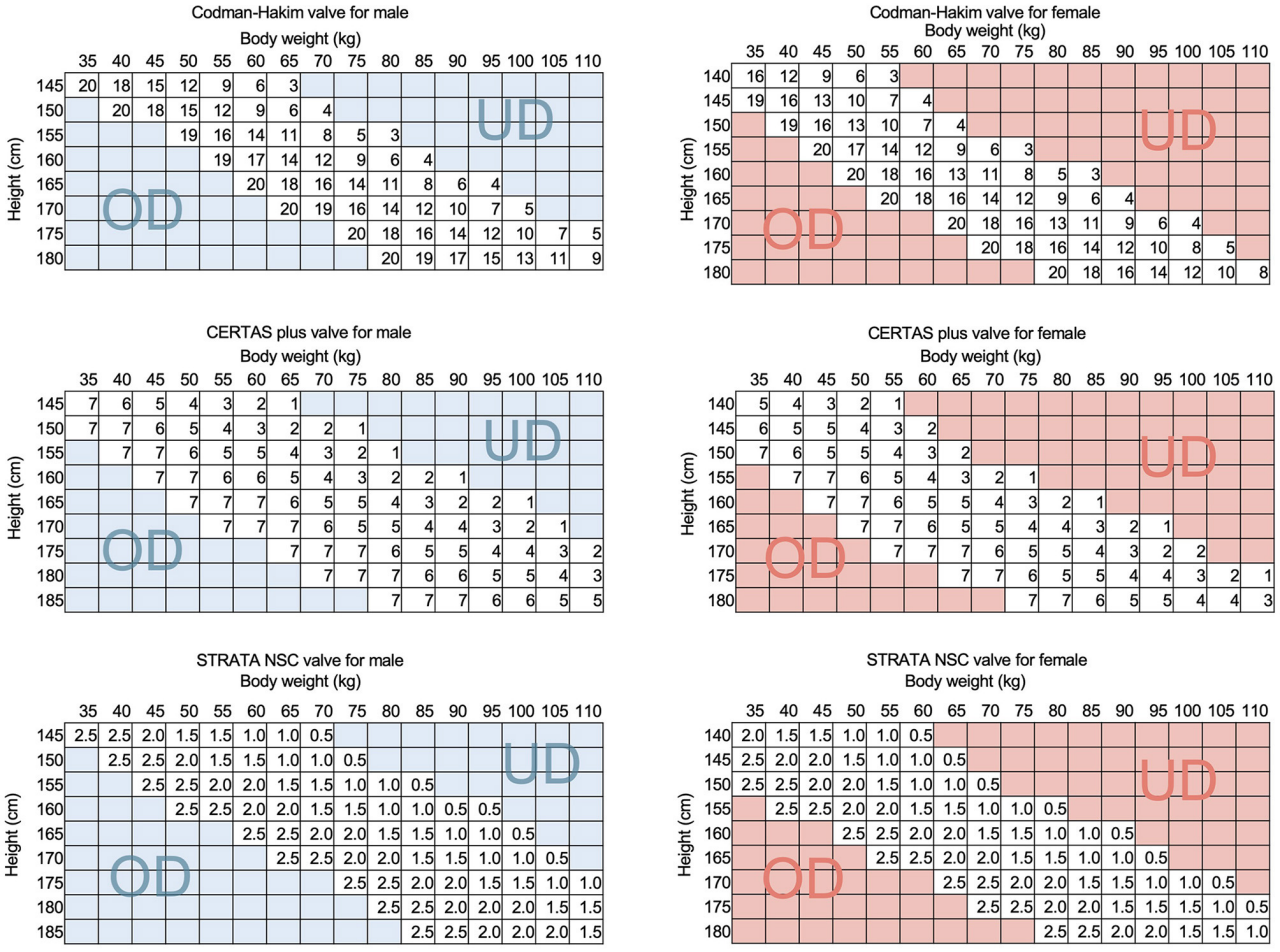
The QRTs of representative programmable valves. The QRTs for CH valve, CERTAS plus valve, and STRATA NSC valve are shown for men and women. The SINPHONI study proved the validity of the initial pressure setting method by QRT by showing a low valve readjustment rate of 44%. This QRT simulates that if a patient's weight increases by 5 kg, the optimal settings of the CH and CERTAS plus valves will be reduced by about 30 mm H_2_O and 1, respectively. This simulation suggests that a weight change of 5 kg is a risk for shunt malfunction. The UD and OD in the figure indicate that patients in this region are at higher risk for underdrainage and overdrainage, respectively. UD, underdrainage; OD, overdrainage.

We have previously reported that the IAP is crucial to the function of the shunt because it offsets 1/3 of the siphoning effect in the ventriculoperitoneal shunt in the upright position (2). Furthermore, we found that the body mass index (BMI) in the sitting position correlated with the IAP (3). These facts indicate that the BMI has a significant impact on shunt function. Sahuquillo et al. also reported that BMI influences VP shunt function *via* IAP (4). The influence of physique on shunt function suggests that the optimal shunt valve pressure can be estimated from physique (3). We clarified the correlation between height and weight on shunt HP and IAP, obtained a formula for estimating the optimal valve pressure from the regression line, and incorporated it into a practical Quick Reference Table (QRT) by the physique (Figure 1) (5). We have proved that the efficacy of this QRT method by the SINPHONI study showed a low resetting rate (6). Currently, the QRT method is the standard for the initial pressure setting after shunt surgery in Japan. These findings provide evidence that the physique is closely related to the shunt function. On the other hand, these facts allow us to raise the hypothesis that the weight gain or weight loss may induce shunt malfunction. We can easily explain this hypothesis from QRT, which shows that a 5 kg change in the body weight can change the optimal valve pressure (Figure 1). However, such shunt malfunctions associated with weight gain and weight loss have not been detected so far, except for those associated with pregnancy (7).

This study is a part of a prospective study on investigating the total pressure environment of a shunt system. We measured the shunt-related pressure, including ICP, when valve setting was required, such as during initial pressure setting or when SM was suspected, then set the valve pressure so that ICP would be within the target pressure range. During the study period, we found five patients with functional SM associated with weight gain or weight loss.

The pressure parameters of the shunt system pressure environment consist of ICP, IAP, hydrostatic pressure (HP), and valve perfusion pressure (PP) in the shunt system (2). The following equation is established between each pressure:


$$\begin{array}{l} PP=\left(ICP-IAP\right)+HP \end{array}$$

In this study, we measured ICP and IAP by puncturing a reservoir implanted in the patient (2). We also estimated the HP of the shunt system from the HP in the extracorporeal tubing passed between the ICP and IAP reference points. Stevin's law states that the pressure at any point in a fluid depends only on the depth of that point. Therefore, the HP on a shunt system can be simplified as the difference in height between two reference points. To measure the height difference between the two reference points, we connected a pressure transducer to a tube filled with water outside the body that passes between the two reference points. The HP can be measured as the water pressure of this height difference. Since the specific gravity of CSF is 1.005 ~ 1.009, the measurement error due to filling the measured tube with water of specific gravity 1.0 is <1%. Thus, we can measure the shunt HP directly as the water pressure from the height difference between the two reference points. The PP of that shunt system, which determines the shunt flow, was defined and calculated by the above equation. These analyses provide deep insights into the mechanism of this new type of shunt malfunction, as well as into normal shunt function. We will also discuss the narrow therapeutic window of valve pressure in patients with NPH and ICP-based shunt management.

## Materials and Methods

The research protocol was approved by the Ethics Committee of Osaka Medical College (No. 27,27-1,2788), and informed consent was obtained in writing from all patients. The 72 patients with shunted normal pressure hydrocephalus (NPH) were prospectively studied at Osaka Medical College Hospital from January 1999 to March 2005 for shunt system pressure environment. During the study period, we found five cases of SM associated with weight change and apparent improvement after valve adjustment. The symptoms of four patients worsened slowly in about 3 months with weight gain. We readjusted the valves, and the gait symptoms improved immediately afterward. We quantitatively confirmed the improvement of the gait symptoms by the 10-m walk test (10MWT). In one patient who lost about 10 kg of weight, the CT revealed an asymptomatic chronic subdural hematoma (CSDH). We readjusted the valve and confirmed that the CSDH had disappeared 1 month later.

### Evaluation of the Total Shunt Pressure Environment

**Figure 2:**
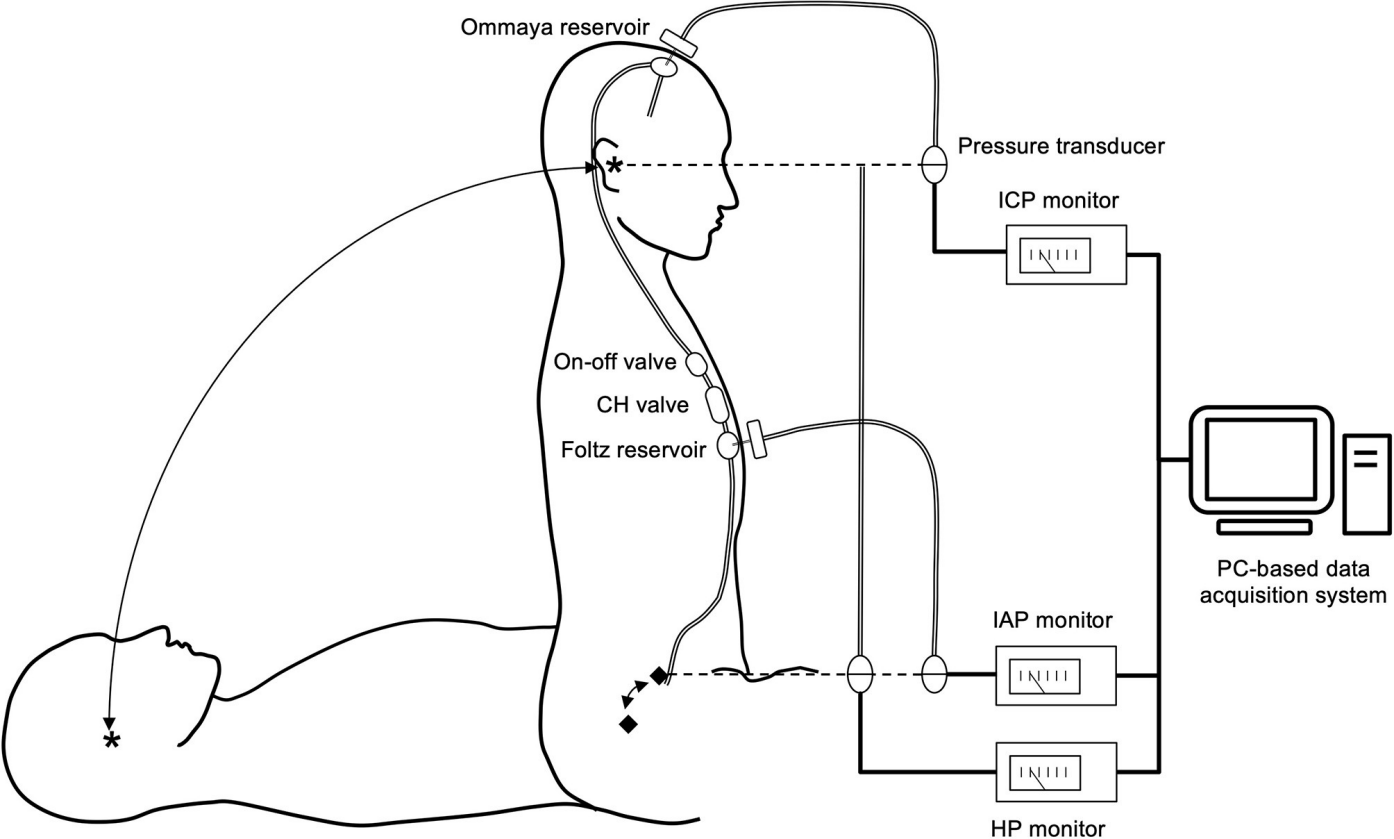
Shunt system and shunt-related pressure measurement system. The patients were implanted with an Ommaya reservoir in the head and an on-off valve, a CH valve, and a Foltz reservoir in the chest. By puncturing both reservoirs with a 22G needle, ICP and IAP can be measured. We defined the reference points for ICP and IAP as the external auditory meatus (*) and the mid-axillary line at the level of the superior anterior iliac crest (♦), respectively. The pressure environment was measured in the supine and sitting positions. We measured the HP of the shunt system as the difference in height between the two reference points. We calculated the shunt PP based on these pressures using the following formula. PP = (ICP–IAP) + HP. Thus, this measurement system provides insight into the comprehensive shunt-related pressure environment. All measurement data were digitized and recorded in a PC-based data acquisition system.

In a study of the shunt system pressure environment, we evaluated the shunt pressure environment of patients during initial pressure setting, after setting, and during shunt malfunction. The patients underwent shunting with a ventricular catheter placed by anterior horn puncture and an Ommaya reservoir (Medtronic Inc, Minneapolis, USA) implanted in the burr hole. In addition, an on-off valve (Integra LifeSciences, Plainsboro, NJ, USA), a Codman–Hakim (CH) programmable valve (Integra LifeSciences, Plainsboro, NJ, USA), and a Foltz reservoir (Integra LifeSciences, Plainsboro, New Jersey, USA) implanted in the right anterior chest wall (Figure 2).

We used the following procedure to assess the total shunt pressure environment. We thoroughly disinfected the skin at the puncture site with povidone–iodine. Then, we punctured the Ommaya and Foltz reservoirs with 22-gauge winged needles and measured ICP and IAP in the supine and sitting positions, respectively, using the pressure transducers connected to these needles. We set the ICP reference point at the external auditory meatus and the IAP reference point at the level of the anterior superior iliac spine at the height of the mid-axillary line. The HP of the shunt system, which is the difference in height between the ICP and IAP reference points, was measured with a pressure transducer. We calculated the PP of the shunt system by the following formula


(1) $$\begin{array}{r} PP=\left(ICP-IAP\right)+HP \end{array}$$

The pressure transducer was connected to the lower end of a semi-open tubing filled with water so that the water surface was at the level of the external auditory meatus and the pressure transducer was at the anterior superior iliac spine. This setup allowed us to directly measure the HP corresponding to the height difference between the ICP and IAP reference points.

The measurement data from all pressure transducers were digitized at 200 samples/second and recorded using a computer-based measurement system. The software for the digital acquisition of data was programmed using the LabVIEW G language (National Instruments Inc., Texas, USA).

### Pressure Evaluation During Initial Pressure Setting and Pressure Setting Based on ICP

In this study of the total pressure environment of the shunt system, we evaluated the shunt system pressure environment after ventriculoperitoneal shunting. With the on–off valve off for 12 h before the measurement, we first evaluated the pressure environment in the preoperative state in the sitting and supine positions. Then, we opened the on-off valve in the sitting position and sequentially lowered the CH valve setting from 200 to 140, 80, and 30 mm H_2_O until the ICP was within the target pressure range. We set this target ICP range from −8 to −13 mm Hg using the external auditory meatus as a reference point and −15 to 20 mm Hg range using the Ommaya reservoir as a reference point. We determined this target pressure range empirically according to preliminary clinical data (2, 3, 8). For each valve pressure change, we recorded for at least 15 min in a sitting position to allow the pressure to stabilize.

### Pressure Evaluation and Management During Shunt Malfunction

When SM was suspected, we measured the pressure environment. We can diagnose mechanical shunt malfunction, i.e., obstruction of the ventricular and peritoneal catheters, from the measured pressure waveforms (8). If the ICP was out of the target pressure range, we readjusted the programmable valve so that the ICP was at the target pressure. If the SM is improved after pressure adjustment, then the shunt pressure environment before pressure adjustment is underdrainage or overdrainage. The pressure environment after pressure adjustment is normal drainage status.

### Statistical Analysis

The data are presented as mean values (± standard deviation). Assessment of ICP levels in patients with normal drainage, underdrainage, and overdrainage was compared in both the supine and sitting positions; differences in ICP between normal drainage and underdrainage were verified by Wilcoxon's signed-rank test. To evaluate the effect of BMI on IAP, a single regression analysis was performed with BMI as the independent variable. A *p* < 0.05 was considered statistically significant. All statistical analyses were performed using JMP pro15 (SAS Institute Inc., Cary, NC, USA).

## Results

### Developmental Course of SM Patients With Weight Change

Four patients had a gradual worsening of gait disturbance over 3 months and an average weight gain of 6.8 kg (Table 1). The head CT scan showed no change in the size of the ventricles as assessed by Evans' index (Table 2). Smooth pumping of the Ommaya and Foltz reservoirs ruled out the possibility of mechanical shunt obstruction. We also confirmed that it was not a mechanical shunt obstruction based on the pulse wave during the shunt pressure environment measurement (8). In one patient with a 10 kg weight loss, a CT scan revealed a thin CSDH without a low-pressure headache.

**Table 1:** Changes in body weight and shunt-related pressures at the time of shunt malfunction.

			**Sitting**		**Supine**	
**Type**	**Case no**.	**ΔBw (kg)**	**ΔICP (mmHg)**	**ΔIAP (mmHg)**	**ΔICP (mmHg)**	**ΔIAP (mmHg)**
UD	1	6	3	11	4.5	4
UD	2	6	5	7	3.5	6
UD	3	6.5	8	10	6	5
UD	4	8.5	4	10.5	0	2.5
	mean ± SD	6.8 ± 1.2	4.8 ± 1.0	8.8 ± 1.6	3.5 ± 2.5	4.4 ± 1.5
OD	5	−10	−5	−5	−4	−2

*This table shows the changes in body weight, ICP, and IAP during the changes from normal drainage to underdrainage with weight gain and overdrainage with weight loss. With an average weight gain of 6.8 kg, the IAP and ICP increased by 4.8 and 8.8 mm Hg, respectively; with a weight loss of 10 kg, the IAP and ICP decreased by 5 kg. The pressure changes were greater in the sitting position than in the supine position*.

*UD, underdrainage; OD, overdrainage; ΔBw, change of body weight; ΔICP, change of intracranial pressure; ΔIAP, change of intra-abdominal pressure; ICP, The units of these pressures are mmHg. These numbers represent mean ± SD*.

**Table 2:** Changes in clinical parameters of WAIST patients.

						**Weight (kg)**		**10MWT (sec)**		**Evans' index**	
**Case No**.	**Type of NPH**	**Age at SM**	**Sex**	**Duration from surgery to SM**	**Types of SM**	**Post OP**	**SM**	**SM**	**After valve readjustment**	**post OP**	**SM**
1	sNPH	78	F	48 M	UD	53	57	29	14	0.296	0.309
2	iNPH	75	M	9 M	UD	50	56	23	15	0.317	0.316
3	iNPH	74	M	28 M	UD	62	68.5	17	10	0.338	0.346
4	iNPH	86	M	8 M	UD	54	60.5	NA	NA	NA	NA
5	iNPH	71	M	21 M	OD	71	61	–	–	0.282	0.216

*WAIST, weight and abdominal pressure-induced shunt trouble; sNPH, secondary normal pressure hydrocephalus; iNPH, idiopathic normal pressure hydrocephalus; SM, shunt malfunction; UD, underdrainage; OD, overdrainage; 10MWT, 10-m walk test; OP, operation*.

### Pressure Changes in Underdrainage Cases Due to Weight Gain

In the four cases of underdrainage, IAP and ICP in the sitting position increased by 8.8 ± 1.6 and 4.8 ± 1.0 mm Hg, respectively, compared with those during normal drainage (Table 1). Table 3 shows the detailed pressures for each case. The body weight increased by 6.8 ± 1.2 kg compared to the postoperative period. As a result, the ICP in the sitting position increased to −6.5 ± 1.9 mm Hg, which was outside the target ICP. In the supine position, the increase in IAP and ICP was limited compared to the sitting position, with 4.4 ± 1.5 and 3.5 ± 2.5 mm Hg, respectively (Table 1).

**Table 3:** Changes in all shunt-related pressures pre-, during, and post-shunt malfunction.

**Case no**.	**Timing of measurement**	**Shunt staus**	**Bw (kg)**	**Ht (m)**	**BMI**	**CH valve (mmH_**2**_O)**			**Sitting**				**Supine**		
							**VP**	**IAP**	**PP**	**ICP**	**HP**	**IAP**	**PP**	**ICP**	**HP**
1	Post OP	ND	53	1.5	23.6	50	3.7	18	7	−13	38	11	−10.5	−1.5	2
	UD	UD	59	1.5	26.2	50	3.7	26	4	−8	38	15	−10	3	2
	After re-setting	ND	59	1.5	26.2	30	2.2	25	1	−12	38	16	−13	1	2
2	Post OP	ND	50	1.5	22.2	120	8.8	14	9	−8	31	6	0	1	5
	UD	UD	56	1.5	24.9	120	8.8	21	6	−4	31	12	−2.5	4.5	5
	After readjustment	ND	56	1.5	24.9	30	2.2	20	4	−7	31	14	−7	2	5
3	Post OP	ND	62	1.65	22.8	160	11.8	8.5	18.5	−12	39	2	1	−1	4
	UD	UD	68.5	1.65	25.2	160	11.8	18	15	−6	39	7	2	5	4
	After readjustment	ND	68.5	1.65	25.2	60	4.4	19	6	−14	39	9	−4	1	4
4	Post OP	ND	52	1.55	21.6	180	13.2	9.5	13.5	−12	35	5	−1.5	1.5	2
	UD	UD	60.5	1.55	25.2	180	13.2	20	7	−8	35	7.5	−4	1.5	2
	After readjustment	ND	60.5	1.55	25.2	150	11.0	20	3	−12	35	7.5	−4	1.5	2
5	Post OP	ND	71	1.71	24.3	60	4.4	18	10	−13	41	5	−4	0	1
	OD	OD	61	1.71	20.9	60	4.4	13	10	−18	41	3	−6.2	−4	1
	After readjustment	ND	65	1.71	22.2	130	9.6	14	16	−11	41	4	−4	−1	1
														(mmHg)	

*The time point of normal drainage status immediately after surgery is denoted by Pre-SM, the time point of shunt malfunction by SM, and the time point of normal drainage after valve readjustment by Post-SM. All pressures are in mm Hg*.

*ND, normal drainage; UD, underdrainage; OD, overdrainage; Bw, body weight; Ht, height; CH valve, Codman Hakim valve; VP, valve pressure; IAP, intra-abdominal pressure; PP, perfusion pressure; ICP, intracranial pressure; HP, hydrostatic pressure*.

**Figure 3:**
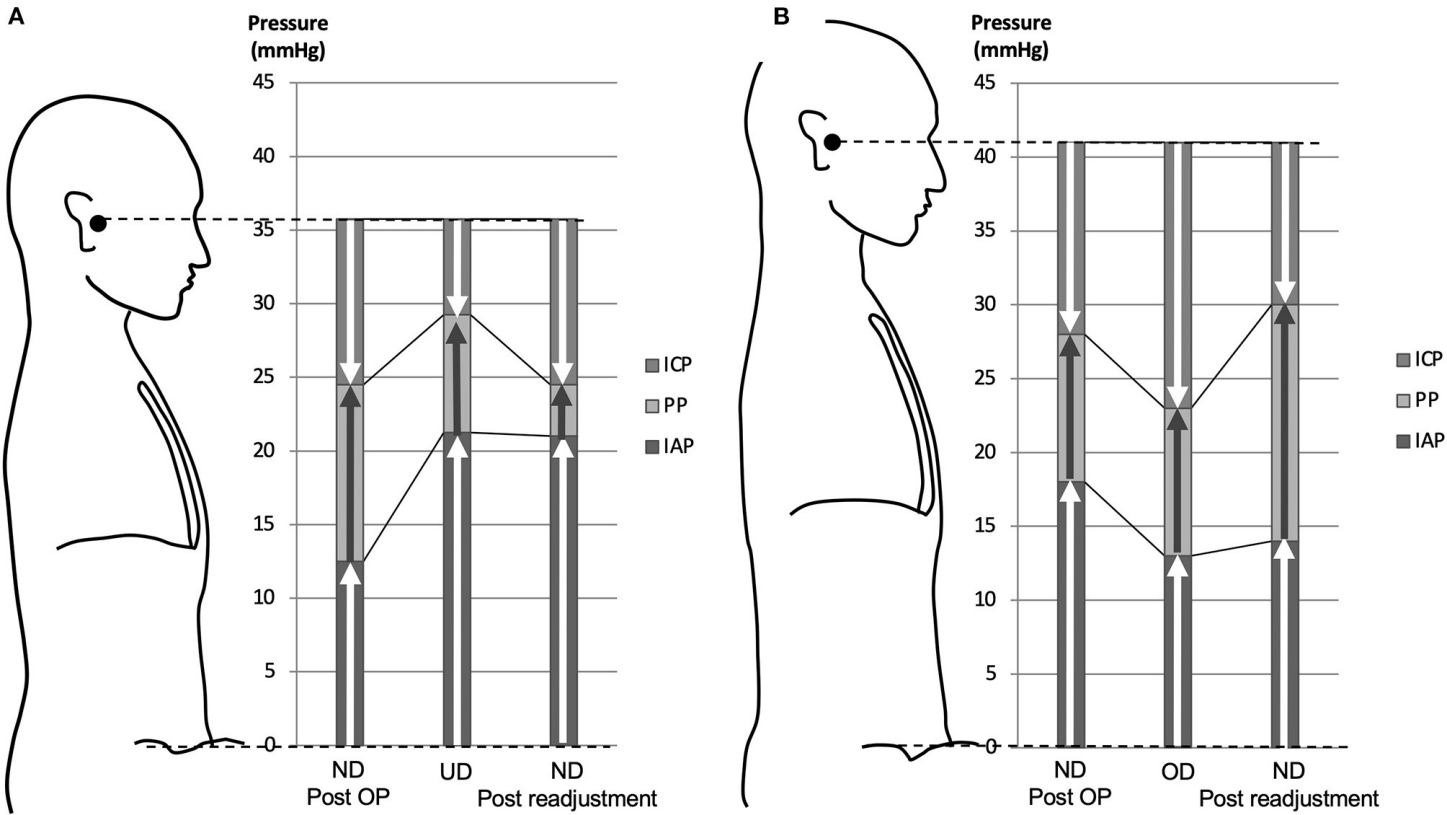
Pressure diagram of underdrainage type WAIST **(A)** and overdrainage type WAIST **(B)**. **(A)** With an average weight gain of 6.8 kg, the IAP increased by 8.8 mm Hg and the ICP increased by 4.8 mm Hg by linking to the IAP. As a result, the ICP is −6.5 mm Hg to the external auditory meatus. This ICP value is greater than the target range of −8 to −13 mm Hg for ICP, which is the underdrainage range. Lowering the valve pressure by 4.4 mm Hg, equivalent to 60 mm H_2_O, reduced the shunt PP, resulting in an immediate recovery of ICP to −11.3 mm Hg, within the target range. **(B)** A 10-kg weight loss reduced IAP by 5.0 mm Hg and ICP by 5.0 mm Hg by linking to IAP. As a result, ICP is −18 mm Hg with reference to the external auditory meatus. This ICP value is lower than the ICP target range of −8 to −13 mm Hg, which is in the overdrainage range. The shunt PP was increased by lowering the valve pressure to 5.1 mm Hg, equivalent to 70 mm H_2_O, and as a result, the ICP was restored to −11.0 mm Hg, within the target range. ND, normal drainage; UD, underdrainage; OD, overdrainage.

By lowering the CH valve pressure by 4.4 ± 3.0 mm Hg (equivalent to 60 mm H_2_O) to resolve the underdrainage, ICP decreased by 4.8 ± 2.2 mm Hg in the sitting position and by 2.1 ± 1.7 mm Hg in the supine position (Table 1). As a result, ICP in the sitting position decreased to −11.3 ± 3.0, recovering to within the target ICP range. On the other hand, IAP was not affected by the readjustment of the CH valve (Figure 3, Table 1). Within 1 h of CH valve readjustment, gait disturbance improved rapidly. In the three cases evaluated in the 10MWT, the average time improved from 23 s before valve resetting to 13 s after resetting.

### Pressure Changes in an Overdrainage Case Due to Weight Loss

**Figure 4:**
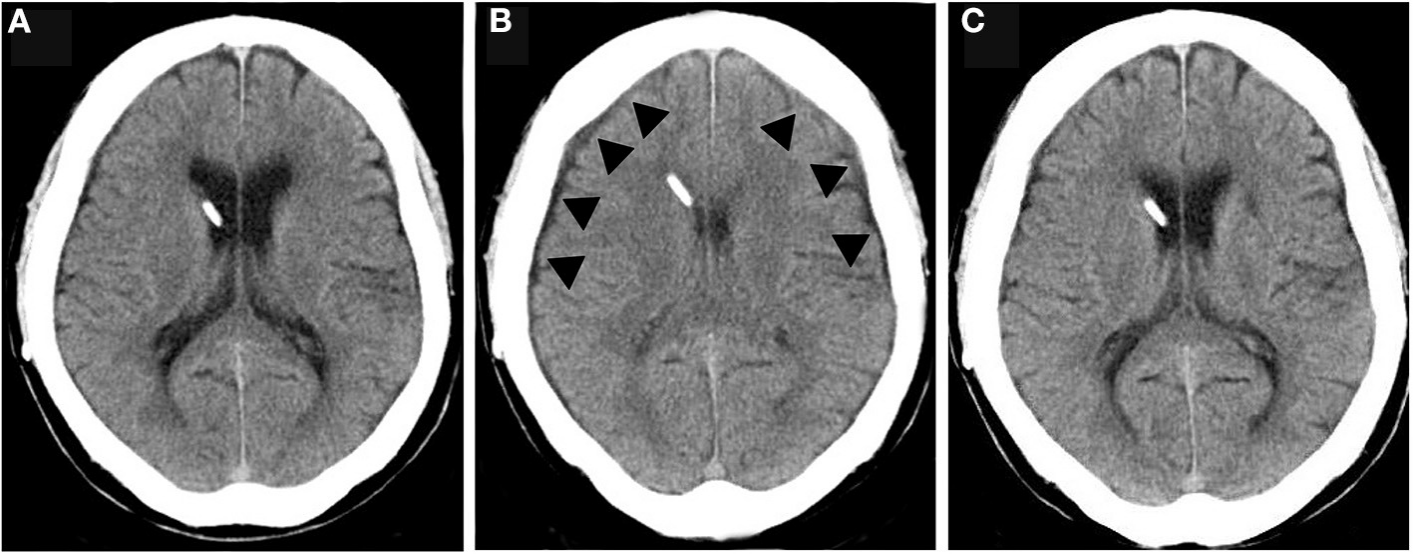
The CSDH caused by overdrainage and disappearance after valve readjustment. **(A)** CT of normal drainage state after shunting; **(B)** CT during overdrainage reveal slit ventricle and thin CSDH (arrowhead); **(C)** CT At 1 month after the valve readjustment shows disappearance of CSDH and slit ventricle.

In one patient, symptoms improved and ventricular size was normal after surgery (Figure 4A). However, at 12 months postoperatively, the patient had lost 10.0 kg of weight, and a CT scan revealed bilateral thin chronic subdural hematomas (CSDH) (Figure 4B). However, the patient did not complain of a low-pressure headache. A weight loss of 10 kg resulted in a −5 mm Hg decrease in IAP and ICP. As a result, ICP decreased to −18 mm Hg, which was below the target pressure. Readjustment of the CH valve increased the ICP to −11 mm Hg, which was in the target pressure range. At 3 weeks after the readjustment, the CSDH spontaneously disappeared (Figure 4C, Table 1).

### Therapeutic Window for ICP in Patients With NPH

**Figure 5:**
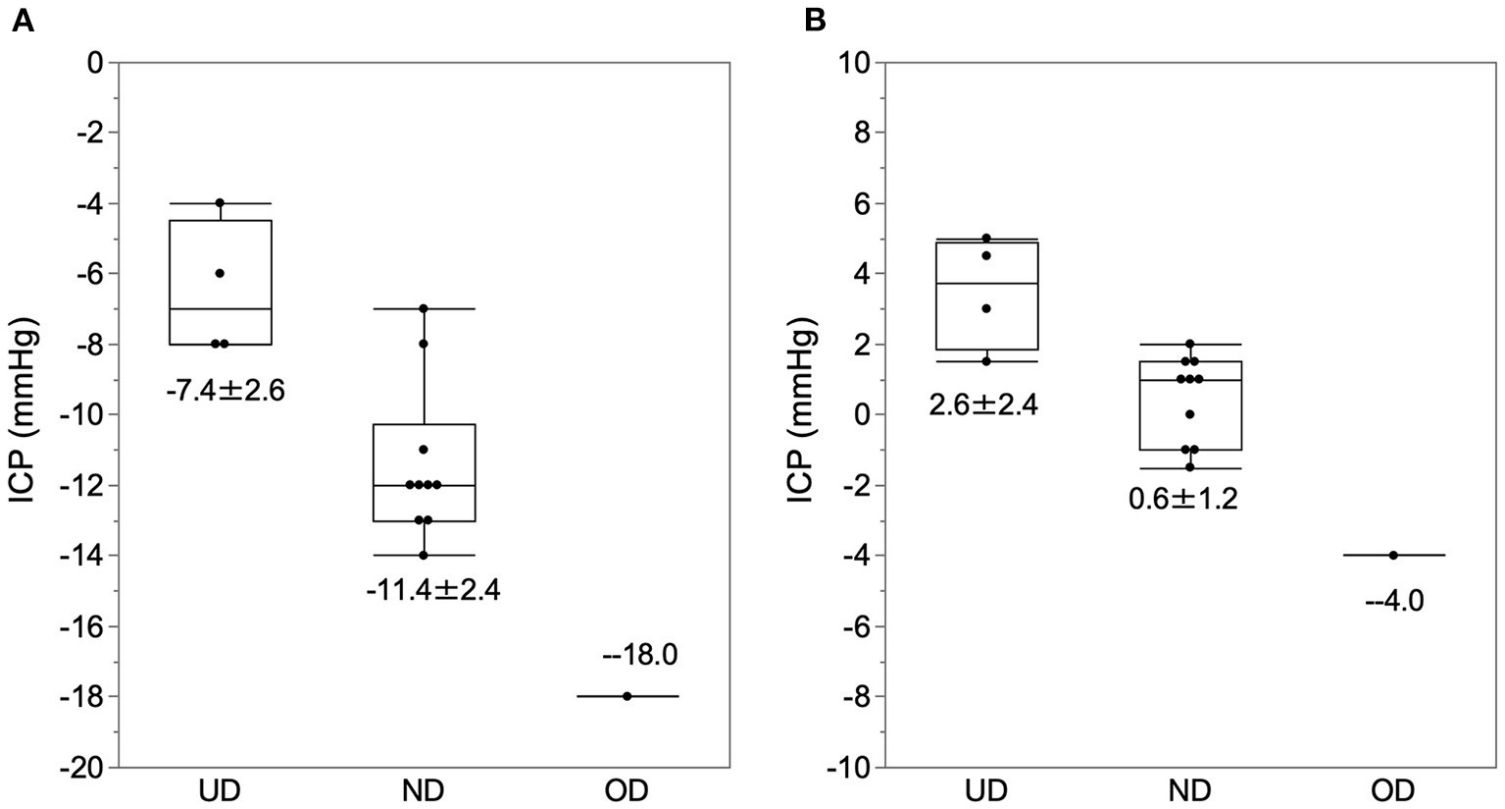
The ICP at the time of normal drainage, underdrainage, and overdrainage. The ICP data at the time of normal drainage, underdrainage, and overdrainage and their boxplots are plotted in the sitting **(A)** and supine **(B)** positions. **(A)** In the sitting position, the ICP for underdrainage, normal drainage, and overdrainage were −7.4 ± 2.6, −11.4 ± 2.4, and −18.0 mm Hg, respectively. (B) In the supine position, ICP levels for underdrainage, normal drainage, and overdrainage are 2.6 ± 2.4, 0.6 ± 1.2, and −4 mm Hg, respectively. The cutoff values of ICP for underdrainage and normaldrainage in the sitting and supine positions were −8.0 and 2.0 mm Hg, respectively. ND, normal drainage; UD, underdrainage; OD, overdrainage.

In the sitting position, the ICP of normal drainage, underdrainage, and overdrainage were −11.4 ± 2.0, −6.5 ± 1.7, and −18 mm Hg, respectively (Figure 5A). In the supine position, the ICP of normal drainage, underdrainage, and overdrainage were 0.7 ± 1.2, 3.5 ± 1.4, and −4 mm Hg, respectively (Figure 5B). There was no significant difference in ICP between normal drainage and underdrainage both in the sitting and supine positions. The threshold pressure between underdrainage and overdrainage in the sitting position was −8 mm Hg, and that in the supine position was 2 mm Hg.

### Correlation and Change Vector Between BMI and IAP

**Figure 6:**
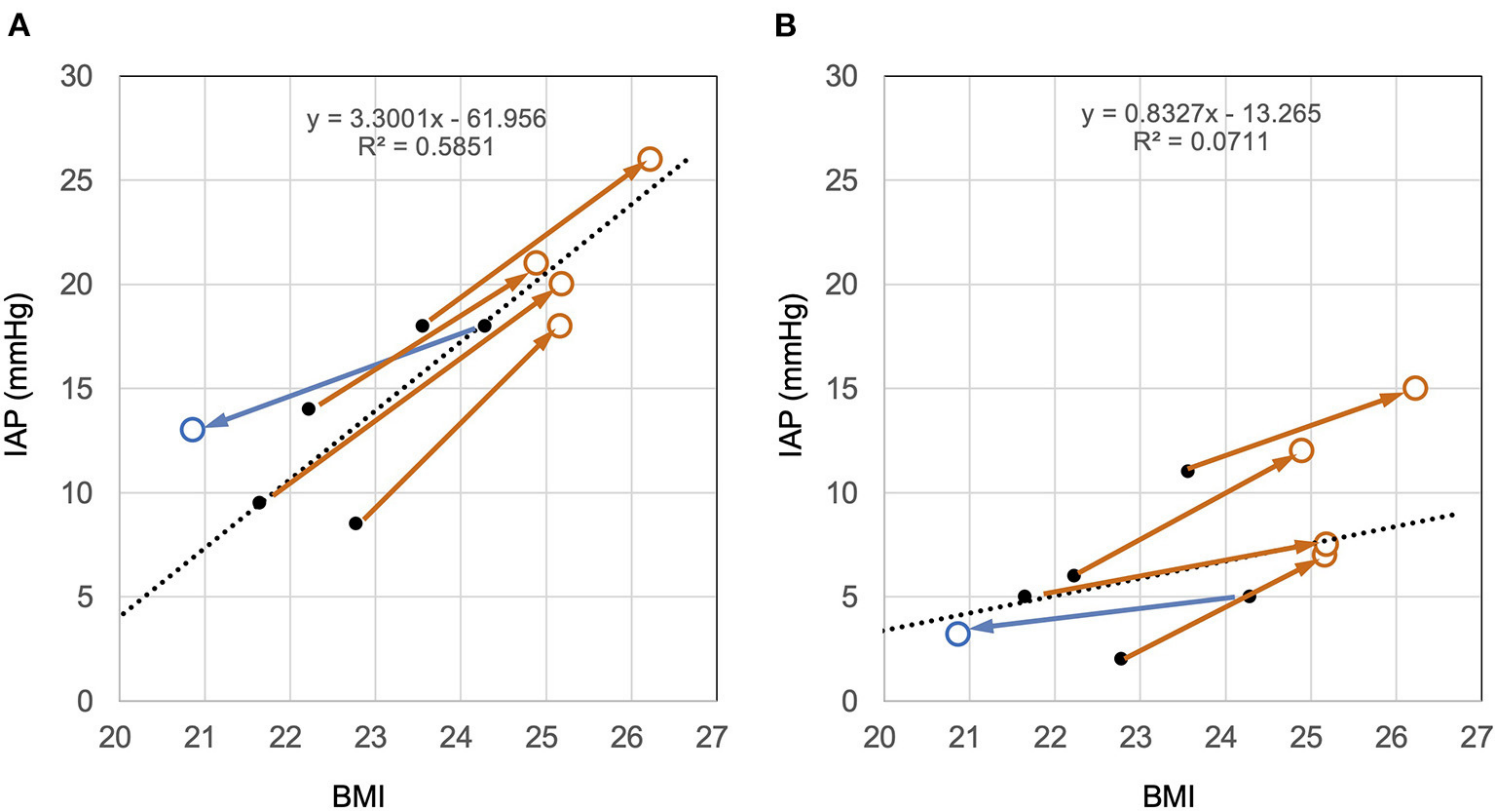
Cross-sectional and longitudinal relationship between BMI and IAP in the sitting **(A)** and supine **(B)** positions. The black dots and dotted lines represent the relationship between the BMI and IAP immediately after surgery and their regression lines, respectively. Brown open circles represent the time point at which the patient gained weight. Blue open circles represent the time of weight loss. **(A)** In the sitting position, IAP immediately after surgery was positively correlated with BMI (dots). The change with weight change is shown as a vector. The slope of the vector and the regression line is similar. This finding indicates that the IAP change with weight change follows the correlation between BMI and IAP. **(B)** The same trend was observed in the supine position as in the sitting position. Judging from the slope of the vectors, the effect of BMI on IAP in the supine position was about half that in the sitting position.

In the sitting and supine positions, BMI was positively correlated with IAP (Figure 6). Pearson's correlation coefficients for the sitting and supine positions were *r*^2^ = 0.47 and 0.49, respectively. The corresponding *p*-values for the sitting and supine positions were *p* < 0.0198 and 0.0077, respectively. The slope of the vector of IAP change in BMI change was almost the same as that of the regression line of BMI and IAP (Figure 6).

## Discussion

### The SM Induced by Changes in Body Weight and Abdominal Pressure

This is the first report to show that weight change can affect shunt function and induce SM in patients with NPH. The IAP, which changed in proportion to the weight change, affected the ICP and consequently induced shunt malfunction. Therefore, we named it weight and abdominal pressure-induced shunt trouble (WAIST). This WAIST consists of the following two types of shunt malfunction: Underdrainage induced by weight gain and overdrainage induced by weight loss.

### The BMI and IAP

This study presented longitudinal data showing that IAP changes proportionally with increasing or decreasing the BMI. The longitudinal data proportional relationship was almost the same as the cross-sectional data. Previously, we reported having a positive correlation between IAP and BMI in the sitting position (2, 3). Many researchers have reported a similar proportional relationship between BMI and IAP (4, 5, 9, 10). However, the limitations of these studies are that they are cross-sectional, with only IAP for the supine position and no data for the sitting position. This study provides a longitudinal demonstration that BMI change in the sitting position affects IAP.

### Linkage Between IAP and ICP in Patients With Shunted Hydrocephalus

We have shown that changes in ICP and IAP are closely related in patients with shunted NPH (Figure 3). Furthermore, we can theoretically prove that these changes are causally related and linked as follows. In the physiological cerebrospinal fluid (CSF) circulation, the passive absorption of CSF into the dural venous system, such as the superior sagittal sinus, depends on the pressure gradient between ICP and venous sinus pressure and CSF outflow resistance. From this relationship, ICP depends on venous sinus pressure and CSF outflow resistance and can be described by the following equation known as Davson's equation (11, 12).


$$\begin{array}{l} ICP=Ro\times If+Pss \end{array}$$

Where Ro is the CSF outflow resistance, “If” is the CSF production rate, and Pss is the sagittal sinus pressure.

A recent study reported that dural lymphatic vessels absorb CSF similar to arachnoid granules (13). Thus, we can describe ICP as follows:


$$\begin{array}{l} ICP=Ro\times If+Pd \end{array}$$

Where Ro is the CSF outflow resistance, “If” is the CSF production rate, and Pd is the dural lymphatic and venous pressure.

On the other hand, in patients with shunted hydrocephalus, most of the CSF outflow is transferred from the physiological CSF absorption system to the shunt system.

When the ICP becomes lower than the venous pressure of the dura mater due to CSF shunting, physiological CSF absorption ceases. The shunt system drains the CSF predominantly.

We can describe the ICP of a patient with shunts in this condition by shunt resistance (Rs) and IAP with the following equation.


$$\begin{array}{l} ICP=Rs\times If+IAP \end{array}$$

Where Rs is the flow resistance of shunt system, “If” is the CSF production rate, and IAP is the intra-abdominal pressure.

In this equation, the ICP of a patient with shunts is a function of the IAP. This equation theoretically explains that the ICP of a patient with shunts links to the IAP.

We have previously proposed that this concept of linkage between IAP and ICP is crucial to the theory of shunt management (3).

### Mechanism of WAIST

**Figure 7:**
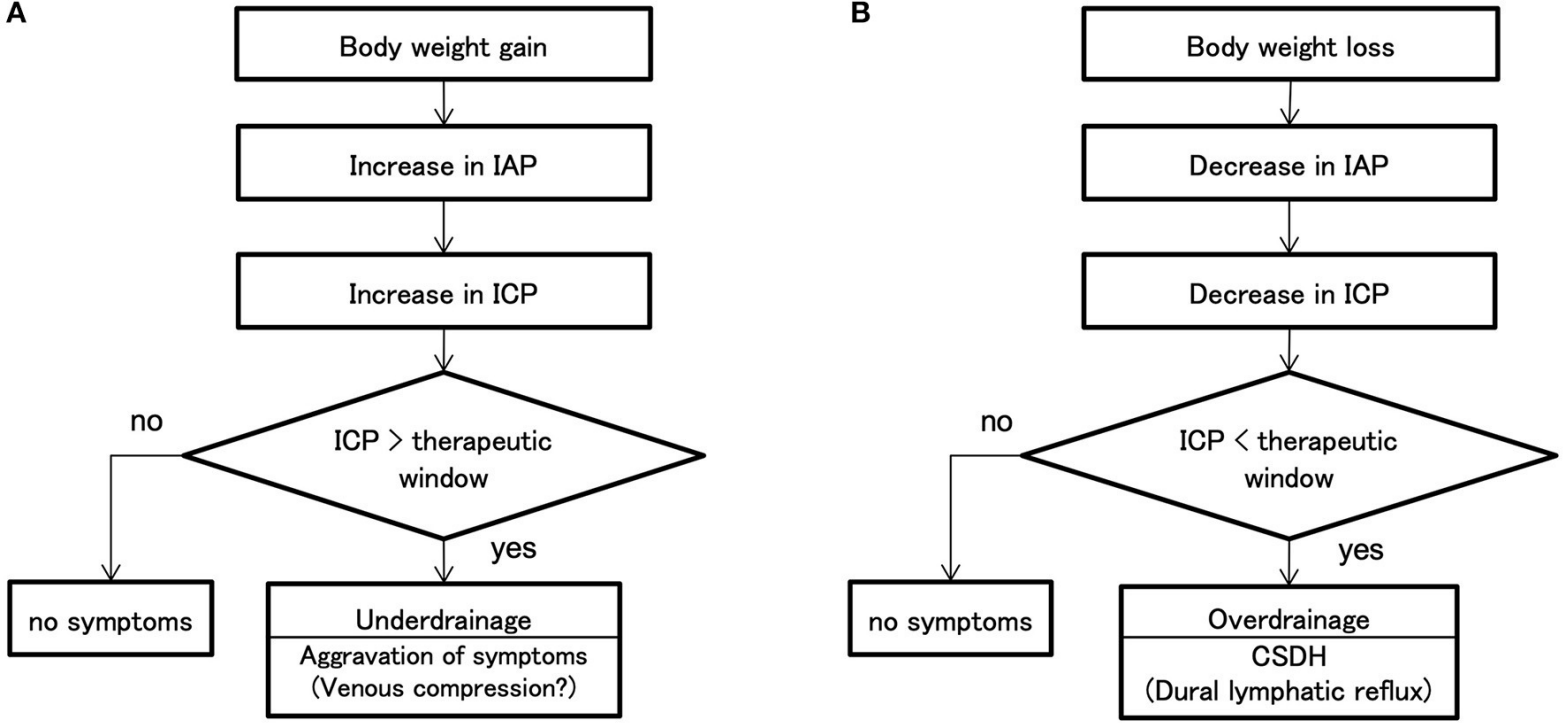
Mechanisms of shunt malfunction associated with weight change. **(A)** Weight gain increases the IAP. In the patients with shunted hydrocephalus, ICP is linked to IAP, so weight gain also increases ICP. Symptoms worsen when the patient's ICP exceeds the therapeutic window. Neurological symptoms with underdrainage are more likely to be cerebral circulatory disturbances due to venous compression because of the immediate improvement. **(B)** Weight loss decreases IAP; ICP linked to IAP decreases simultaneously; if ICP falls below the therapeutic window, a CSDH occurs. If the patient's ICP falls below the therapeutic window, CSDH develops. In this study, we showed that at the time of overdrainage, the subdural space has a significant negative pressure of −18 mm Hg against intradural veins and lymphatic vessels. Since there are few valves in the lymphatic vessels of convexity dura, the backflow of lymphatic fluid in the lymphatic vessels may lead to lymphatic effusion in the subdural space. ICP, intracranial pressure; IAP, intra-abdominal pressure; CSDH, chronic subdural hematoma.

Based on these findings, we can propose the following mechanism for underdrainage type and overdrainage type WAIST (Figure 7). The starting point of underdrainage type WAIST is an increased IAP due to a weight gain of several kilograms (Figure 7A). Then, linked to the increase in IAP, ICP also increases. When the ICP exceeds the upper limit of the therapeutic window, cerebral circulatory disturbances result from compression of the capillaries and small veins of the brain (14), which leads to worsening of the symptoms of NPH. The immediate improvement in symptoms after valve readjustment suggests the involvement of cerebral circulatory disturbances.

In the overdrainage type WAIST, the starting point is a decrease in IAP linked to a weight loss of several kilograms (Figure 7B). ICP also decreases, linked to the decrease in IAP. As ICP falls below the lower limit of the therapeutic range, the convexity level ICP drops to about −25 mm Hg. This negative ICP exposes the medial side of the dura to significant negative pressure. The pressure in the dural venous sinus in the upright position is at the foramen magnum level, approximately the same as in the external auditory meatus, and at atmospheric pressure. Thus, a significant pressure gradient of −18 mm Hg occurs in the thin region between the intradural veins and lymphatic vessels and the subdural space. Since there are few valves in the dural lymphatics in the upper skull (13), lymphatic fluid from the dural lymphatics can easily flow backward, and this becomes the subdural fluid collection. We call this the dural lymphatic reflux hypothesis.

### Similarities and Differences With SM in Pregnant Women

There have been several reports of shunt failure associated with underdrainage in pregnant women. However, researchers have believed that the cause of underdrainage is the elevated position of the peritoneal catheter (15, 16). In the hydraulic model of the abdomen, the abdominal cavity is a single fluid compartment whose density is approximately equal to that of water (2). Therefore, the position of the shunt tube does not affect the PP of the shunt system.

On the other hand, as the abdominal volume of a pregnant woman increases, the IAP increases (1). Therefore, the true cause of underdrainage in pregnancy is not the position of the peritoneal catheter, but the elevated IAP (7). Thus, the mechanism for underdrainage due to weight gain and underdrainage in pregnant women is almost the same. However, the increase in abdominal pressure in pregnant women reaches 40 mm Hg in the sitting position, while the IAP in patients with underdrainage type WAIST is only half of that (17, 18). The reason why underdrainage type WAIST is more sensitive to a slight increase in IAP than underdrainage during pregnancy may be related to the narrow therapeutic window of NPH, which will be discussed next.

### The Therapeutic Window of ICP in Patients With Shunted NPH

The optimal therapeutic window for ICP in patients with shunts with NPH is still unknown. Based on our preliminary studies, we assume that the therapeutic window for ICP is in the range of −8 to −13 mm Hg, with the external auditory meatus as the reference point (2, 3, 8). Although the number of WAIST cases in our study was small, the ICP of normal drainage in the sitting position was almost the same as the target ICP range, with an upper limit of the therapeutic range of −6.5 mm Hg and a lower limit of about −16 mm Hg (Figure 5). However, we can verify the validity of this therapeutic window by analyzing more cases in the comprehensive study on pressure environment of shunt system.

Despite the slight ICP difference between underdrainage and normal drainage in the sitting position, it resulted in a critical difference in clinical symptoms. The reason for this is unclear, but minor compression of the cerebral venous system may affect cerebral circulation (14), and we have found evidence to support this in another study (unpublished).

### Prevention and Treatment of WAIST

The following two things are essential to prevent this WAIST: First, neurosurgeons need to tell patients and their families to maintain their weight at the initial pressure setting. Second, the physician should monitor the patient's weight at follow-up. The patients should maintain their weight or let their physician know if they are planning on losing or gaining weight. If the patient's weight has increased by several kilograms, the physician should evaluate for exacerbation of symptoms such as gait disturbance. If there is an exacerbation of obvious NPH symptoms with weight gain, then the underdrainage type WAIST should be considered first. The physician should lower the valve pressure by 30–60 mm H_2_O and quantitatively compare the gait function before and after valve resetting by a gait function test such as the 3-m Timed Up and Go Test. If the symptoms improve with valve readjustment, the physician should fix the pressure setting. The physician should also check the subdural fluid collection by CT scan after 1 month. On the other hand, if a patient with shunted NPH loses a few kilograms of weight, the physician should check for the development of CSDH on a CT scan. If CSDH appears, the physician should increase the valve pressure in the range of about 30–60 mm H_2_O. At 1 month after the valve readjustment, the physician should check the size of the subdural hematoma with a CT scan.

### Incidence of WAIST and the Reasons for Missing It

Even though mechanical SM did not occur during this study, WAIST occurred in five patients. This fact suggests that WAIST is not rare. Recently, Gutowski et al. (19) reported that 20% of patients with NPH had secondary deterioration during a mean follow-up of 2.7 years, of which 26% improved after valve reconfiguration. Although their report does not describe weight change, WAIST is likely to be the primary cause of that secondary deterioration.

There are two possible reasons for the lack of recognition of WAIST as discussed in the following: The first reason is that CT and shuntography cannot identify WAIST. CT scan and shuntography can easily show mechanical SM as a change in ventricle size or obstruction of the shunt system (1). However, in the case of underdrainage type WAIST, the shunt system is not mechanically obstructed and the size of the ventricles does not change. The second reason is that the symptoms progress slowly and over several months. Physicians tend to attribute the slow progression of symptoms in patients with shunted NPH to aging or other comorbidities.

### Diagnostic Processes for Symptom Aggravation

**Figure 8:**
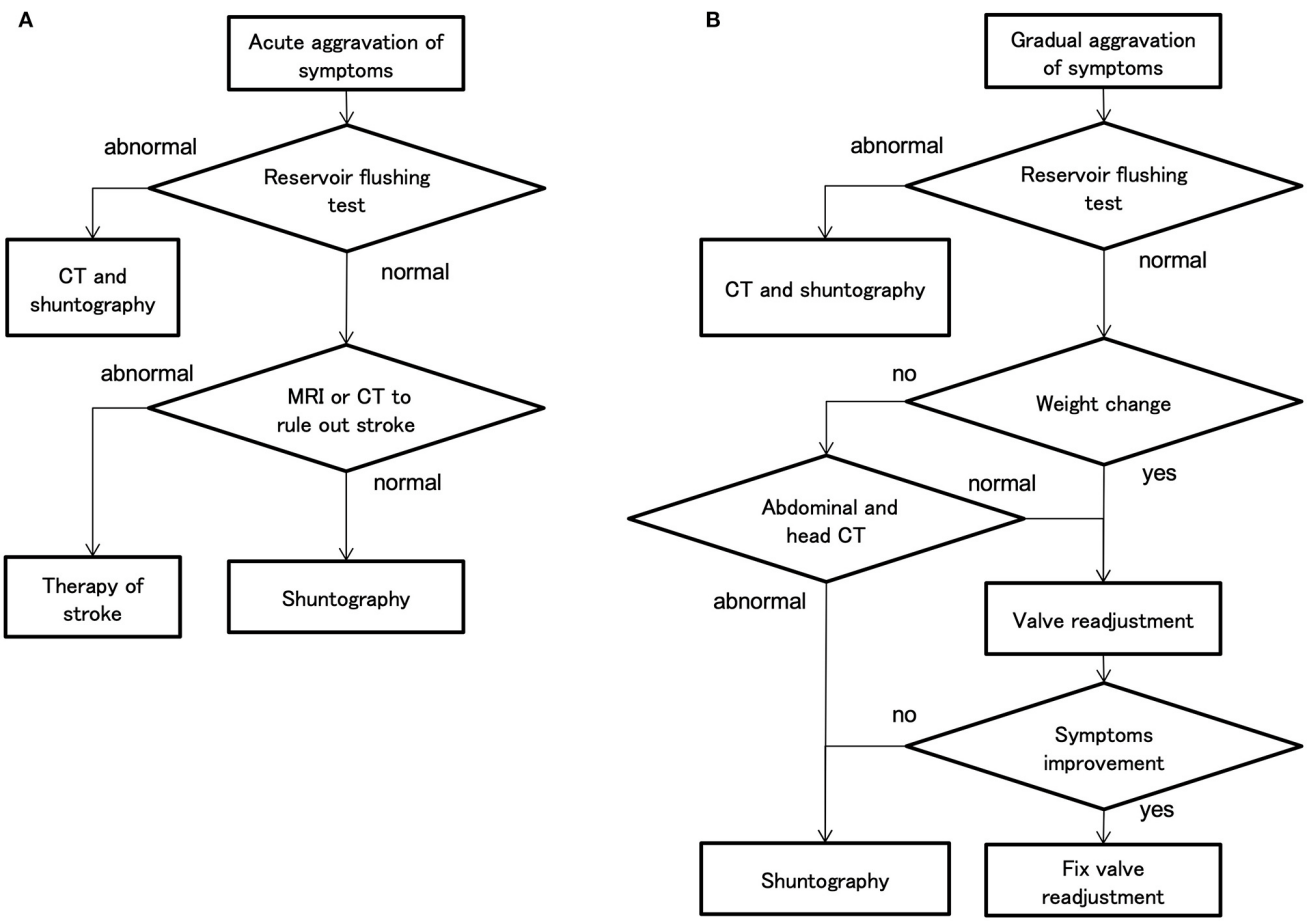
The decision flow chart at symptom aggravation. **(A)** In cases of acute aggravation, the first step is to perform a reservoir flushing test, which can be easily performed in the examination room. If the reservoir flushing test is normal, the physician should differentiate a stroke by magnetic resonance imaging (MRI). **(B)** In cases of gradual aggravation in about 3 months, first perform a reservoir flushing test. If the reservoir flushing test is normal and the weight has changed by several kilograms, the physician should readjust the valve. In the case of weight gain, lower the setting by 30–60 mm H_2_O for the CH valve and by one level for the CERTAS plus and STRATA NSC valves. If there is no improvement after 1 month of valve readjustment, the physician should consider performing shuntography.

In the case of acute aggravation, the decision process is simple because there are only a few differentiating diseases, such as mechanical shunt dysfunction and stroke (Figure 8A). However, in the case of gradual aggravation, there are many differentiating pathologies: mechanical shunt malfunction, WAIST, secondary deterioration (19), musculoskeletal disease, disuse dysfunction, and aging (Figure 8B). As a result, the diagnostic process is complicated (Figure 8B).

The physician must also determine whether the patient's complaints of aggravation are truthful. Therefore, the physician must prove the aggravation of symptoms by objective evaluation. Popular assessment methods are 3-m timed up and go and 10MWT for gait, and MMSE for cognition. The physician should perform these objective assessments routinely and regularly during outpatient follow-up. Weight should also be routinely measured for WAIST evaluation. Performing these assessments requires multidisciplinary collaboration among physical therapists, speech therapists, and nurses.

If the symptoms aggravate gradually over a period of about 3 months, the first step is to perform the reservoir flushing test. This test is a simple and highly accurate detection of proximal catheters, i.e., ventricular and lumbar catheters, and valve obstruction (20). If the reservoir flushing test is normal and the patient's weight has changed by a few kilograms, the physician should first attempt to readjust the valve. In the case of aggravation of gait disturbance by WAIST, improvement of symptoms can be observed by objective gait assessment within 1 h after valve readjustment. However, if symptoms have not improved after 1 month, the physician should consider performing shuntography. If shuntography reveals abnormalities, i.e., occlusions, ruptures, disconnections, perform appropriate shunt revisions (21). However, such multidisciplinary cognitive and gait assessment, and diagnosis by CT and shuntography, are very tedious and consume a lot of medical resources.

Recently, the implantable ICP sensors have become commercially available, and the measured ICP can qualify the drainage status of the patients with hydrocephalus (8, 22). This study also provides evidence that ICP acutely reflects drainage status. Thus, an ICP-based patient management system using an implantable ICP sensor would simplify patient management. It would also be possible to reduce most of the management costs of the patients with shunted hydrocephalus.

## Conclusions

We report a SM induced by changes in body weight and IAP in the patients with shunted NPH and named it WAIST. In underdrainage type WAIST, weight gain exacerbated the symptoms, while in overdrainage type WAIST, weight loss induced CSDH. Both are reversible by the readjustment of the valve. This study presents that weight change affected the IAP and caused shunt malfunction by altering the ICP linked to the IAP. This study recommends that physicians should monitor for changes in weight and symptoms in patients with shunted NPH.

## Data Availability Statement

The datasets generated for this study are available on request to the corresponding author.

## Ethics Statement

The research protocol was approved by the Ethics Committee of Osaka Medical College (No. 27,27-1,2788) and informed consent was obtained in writing from all patients.

## Author Contributions

YK and HM made substantial contributions to the conception and design of the study. YK and MKamo collected data regarding the participants and task performance. MKamo, YK, and TO analyzed the data. MKamo, YK, MW, MKame, and AT wrote the manuscript. All authors read and approved the submitted version.

## Funding

This research was partially supported by Grant-in-Aid for Scientific Research (B) No. 10470297 from the Japan Society for the Promotion of Science.

## Conflict of Interest

The authors declare that the research was conducted in the absence of any commercial or financial relationships that could be construed as a potential conflict of interest.

## Publisher's Note

All claims expressed in this article are solely those of the authors and do not necessarily represent those of their affiliated organizations, or those of the publisher, the editors and the reviewers. Any product that may be evaluated in this article, or claim that may be made by its manufacturer, is not guaranteed or endorsed by the publisher.
